# Phylogeography of bivalve *Meretrix petechialis* in the Northwestern Pacific indicated by mitochondrial and nuclear DNA data

**DOI:** 10.1371/journal.pone.0183221

**Published:** 2017-08-16

**Authors:** Xiaoxuan Wang, Lingfeng Kong, Jun Chen, Akihiko Matsukuma, Qi Li

**Affiliations:** 1 Key Laboratory of Mariculture, Ministry of Education, Ocean University of China, Qingdao, China; 2 Institute of Geology and Paleontology, Linyi University, Linyi, China; National Cheng Kung University, TAIWAN

## Abstract

The marine clam *Meretrix petechialis* is an important economic shellfish species in Northwestern Pacific, but little is known about its phylogeographical pattern. Here, we analyzed 311 samples from 22 locations along the northwestern Pacific using combined profiling of one mitochondrial gene (the first subunit of cytochrome coxidase, COI) and one nuclear DNA marker (the internal transcribed spacer region 1, ITS-1) to investigate contemporary genetic structure and reconstruct phylogenetic history of this species. The results revealed that two distinct phylogeographic lineages dominated marginal seas—the East China Sea (ECS) and the South China Sea (SCS) respectively. The estimation of divergence time between two lineages was 2.1–3.8 Ma, corresponding to a period of the early Pleistocene to late Pliocene. The vicariance of the two lineages was connected to the historical isolation of marginal seas and sea surface temperature (SST) gradient, pointing that SST might play an important role in maintaining phylogeographical patterns of *M*. *petachialis*. Significant overlaps between two lineages were observed in 23° to 29° N, located at the adjacent area of the ECS and SCS, which might be promoted by the connectivity of China Coast Current. However, the influence of ocean currents on mixings between two lineages was limited. In comparison, significant relationships were found between genetic distances and geographic distances if the North and South populations were analyzed separately, result of which might be due to some small reciprocal, rotating flows along coastal areas and special geographical conditions.

## Introduction

Historical climatic changes in sea level, water temperature and ocean currents can have a powerful impact on the distribution and genetic structure of the marine biota [1,2]. During the Pleistocene, glacial-interglacial climate fluctuations resulted in falling and rising sea levels that alternately exposed and submerged shallow seafloors [3], which in the end might have led to genetic divergence and demographic expansion, respectively [4,5]. As an important marine centre of origin, the Northwest Pacific [6], especially China Seas, have gained much attention to understand the past global climate and marine biogeography. China Seas have a long coastline across the temperate, subtropical and tropical climate zones. From the north to the south, the number of species in China Seas increases distinctly [7]. China Seas were separated into two marginal seas: the East China Sea (ECS) and the South China Sea (SCS), connected each other by the Taiwan Strait (<100 m depth). During glacial epoch, according to Miller et al., the major sea-level dropping 60 to 120 m started in Late Pliocene (~2.5 Ma ago) [8], so that land bridges were formed between Asian continent and Taiwan Strait, which isolated two marginal seas to make ECS and SCS serve as two independent glacial refugia [9,10]. During the interglacial period, Taiwan Strait became flooded as the sea-level rose, and then the barrier of ECS and SCS was disappeared. Thereafter, historical population demographic expansion might occur in marine organisms inhabiting in the ECS and SCS.

Sea surface temperature (SST) is always an important factor governing dispersal range of marine organisms [11]. Studies have shown that the range limits of some bivalve species were mainly affected by SST gradients [12,13]. The intraspecific phylogeographic patterns frequently correlate with interspecific biogeographic patterns in the Indo-Pacific or in other marine regions [14–16]. As a barrier, thermal gradients in SST may limit the dispersal of pelagic larvae, and it has been considered to be a plausible driver for the species diversification, such as the flathead mullet *Mugil cephalus* in northwestern Pacific [17], the supralittoral isopod *Ligia occidentalis* in North America [18], and the marine clam *Lasaea australis* in Australia [19]. Furthermore, SST could be affected by sea level fluctuation. In glacial epoch, sea level falling caused the closure of the Japan Sea, so that the warm Tsushima Current, a branch of the Kuroshio Current was prevented from entering the Japan Sea, leading to rising temperature of northern areas [20].

**Fig 1:**
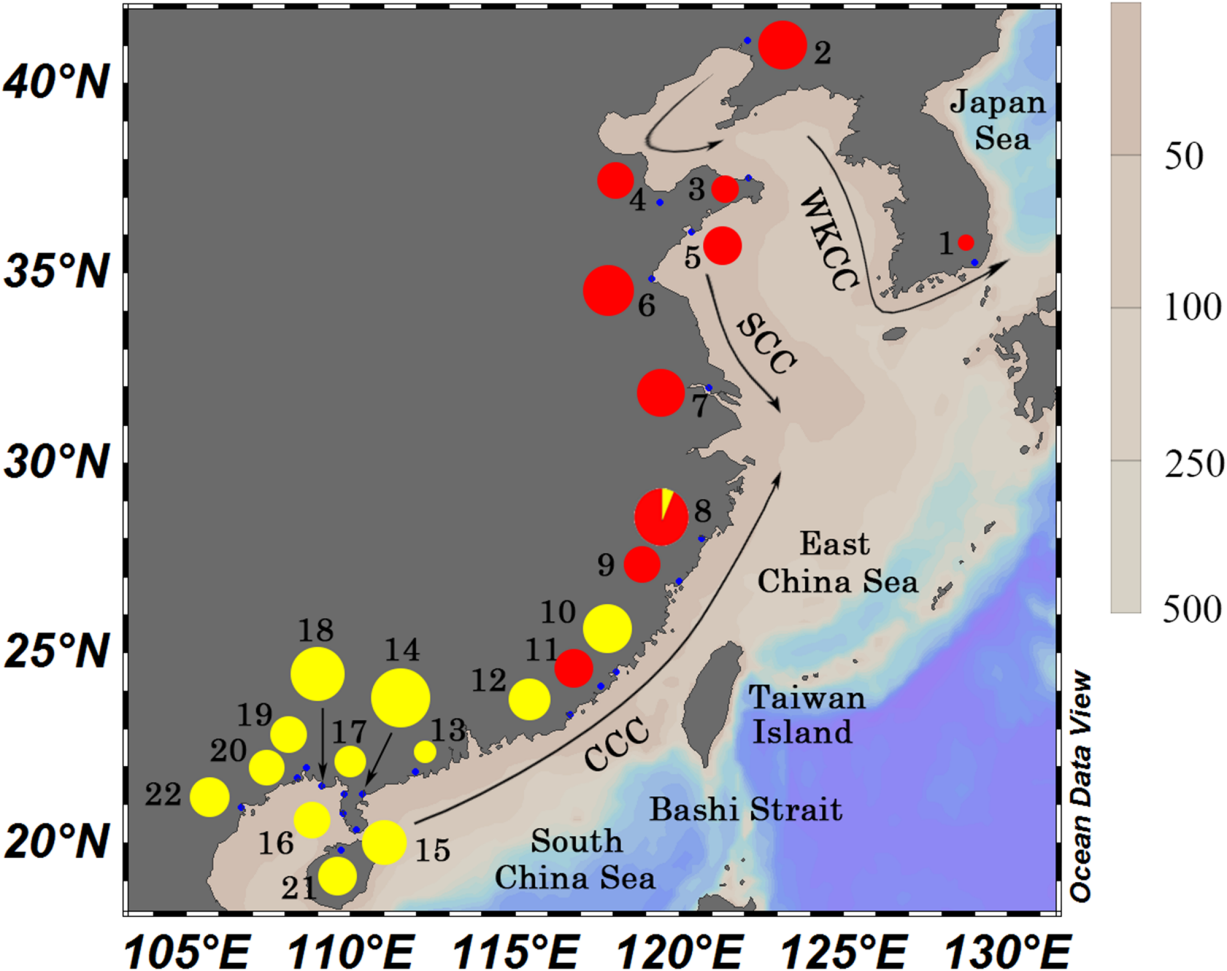
Map showing sample locations, spatial distribution of the two mitochondrial clades by pie diagrams for *M*. *petechialis* and the coastal currents in summer. Sampling sites are labelled with numbers in Table 1. Two lineages are color-coded separately: Red, North lineage; Yellow, South lineage. CCC, China Coastal Current; WKCC, West Korea Coastal Current; SCC, Subei Coastal Current. This map is rendered with ODV v4.7.6 [35].

Ocean currents have an important influence on secondary contract by promoting larval dispersal and enriching population connectivity [21–23]. In China Seas, two currents: the warmer China Coastal Current (CCC) flowing from the SCS into the ECS through the Taiwan Strait, which transports a large amount of warm-water marine species from their tropical center to the north [7] and the colder Subei Coastal Current (SCC) flowing southward along the ECS coast (Fig 1) in summer, which would potentially play a significant role in enhancing larval dispersal in many marine species [24–26]. At the same time, West Korea Coastal Current (WKCC) flowing southward along the Korean Peninsula through the Tsushima Strait enters the Japan Sea. Generally speaking, pelagic larval stage of marine species vary from several days to months, and an extended pelagic larval stage increases the opportunities for long-distance dispersal in the marine realm and is often associated with little genetic differentiation over large geographical distances [27]. However, genetic patterns are not simply related to pelagic larval duration, but also compounded with other factors, e.g. fecundity, life histories and sea temperature [28–30]. Furthermore, sea surface temperature can be affected by ocean currents. Under the influence of warm currents, the water temperature is relatively high, while cold currents make water temperature decrease [7]. To date, a small number of studies have been conducted in shellfish to elucidate the phylogeographic patterns in the coastal areas of China, including the bivalve *Cyclina sinensis* [31], *Ruditapes philippinarum* [32] and *Tegillarca granosa* [33], rock shell *Thais clavigera* [26], the limpet *Cellana toreuma* [34]. However, the phylogeographic patterns of most mollusk species have not been well characterized yet.

*Meretrix petechialis* is an important economic shellfish species, widely distributed along the coastal areas of the northwestern Pacific, as far north as the Bohai Sea, and as far south as Beibu Gulf. The water temperature in reproductive period for *M*. *petechialis* was 20–30°C [36]. This eurythermic clam inhabits the tidal flats, estuaries and sandy beaches [37] with a relative short pelagic larvae stage duration of 5–8 days before settlement and metamorphoses. *M*. *petechialis* was able to live in a salinity ranging from 17.3 to 28.4‰ in incubation period, 9.2 to 29.3‰ in pelagic larval stage and 9.2 to 36.0‰ in larval period, and was more successful in lower salinity environments [38]. Culture of *M*. *petechialis* has been developed rapidly to meet the huge demand of market, and consequently this clam had become one of the most commercially maricultured bivalves in China. In order to ensure sustainable exploitation, management and conservation must rely on population genetic structure and gene flow among populations of the species [39]. *M*. *petechialis* was often misidentified as *M*. *meretrix*. A previous study reported that *M*. *meretrix* was only distributed in the South China Sea, while *M*. *petechialis* was more widely distributed throughout the coasts of China [40]. Genetic surveys of *M*. *petechialis* populations have been performed in China Seas [41,42], however, sampling locations were limited in these previous studies and no accurate factors that affected the genetic population structure were elucidated.

Mitochondrial DNA is well established to study population genetics and phylogeography due to its comparatively fast rate of evolution and lack of recombination [43]. Because of ease of amplification using the polymerase chain reaction method and universal primers [44], COI (the first subunit of cytochrome coxidase) is one of the most frequently used mitochondrial genes and has been widely applied to a number of phylogeographical studies for marine species including *Ruditapes philippinarum* [32], *Cellana toreuma* [34], the scyphozoan jellyfish Aurelia spp. [45] and the Rock Shell *Thais clavigera* [26]. MtDNA markers are maternally inherited, representing only one evolutionary history [46]. Combining nuclear and mitochondrial DNA markers can improve the power of molecular data to test phylogeographic hypotheses and provide complementary information about population structure [25,47]. ITS1 gene (the internal transcribed spacer region 1) is a common nuclear marker used with COI gene in phylogeographic studies [30,31,33]. In the present study, we used COI and ITS1 markers to determine phylogeographical pattern of *M*. *petechialis* populations in the northwestern Pacific.

This work aims to (1) understand the contemporary genetic structure of *M*. *petechialis*; (2) calculate approximate divergence time between major lineages; (3) elucidate the factors that may affect the genetic population structure.

## Materials and methods

### Sample collection

A total of 311 *M*. *petechialis* samples were collected from 22 locations along the coastal areas of China, Korea and Vietnam (Fig 1) during 2006–2016 (Table 1), and were preserved in 95% ethanol. Specific permit was not required to collect *M*. *petechialis* from these locations/activities, because *M*. *petechialis* is not an endangered or protected species and were only collected from public access areas.

**Table 1:** Number of individuals sampled per site (N, parentheses: total number of sequences included), number of haplotype (n), haplotype diversity (h), nucleotide diversity (π), and mean number of pairwise differences (k) were shown for each population. Subscripts indicate variables for COI or ITS-1. Abbr, site abbreviation.

Sampling site		Province/Country	Coordinates	Abbr.	COI					ITS				
					*N*_*C*_	*n*_*C*_	*h*_*C*_	*π*_*C*_	*k*_*C*_	*N*_*I*_	*n*_*I*_	*h*_*I*_	*π*_*I*_	*k*_*I*_
1	Busan	Korea	35°16' N, 128°59' E	BS	2	1	0.0000	0.00000	0.00000	2(7)	7	1.0000	0.00792	6.58873
2	Panjin	Liaoning	41°7' N, 122°4' E	PJ	19	9	0.8713	0.00232	1.73166	4(8)	7	0.9643	0.00739	6.13667
3	Weihai	Shandong	37°31' N, 122°7' E	WH	6	6	1.0000	0.00332	2.47957	1(2)	2	1.0000	0.00370	3.02250
4	Changyi	Shandong	36°51' N, 119°24' E	CY	11	5	0.7091	0.00196	1.46402	4(17)	14	0.9706	0.00858	7.13988
5	Qingdao	Shandong	36°4' N, 120°23' E	QD	12	10	0.9545	0.00321	2.39871	5(14)	14	1.0000	0.00871	7.24128
6	Ganyu	Jiangsu	34°50' N, 119°11' E	GY	21	9	0.8524	0.00234	1.74524	4(13)	12	0.9872	0.00852	7.17581
7	Nantong	Jiangsu	31°59' N, 120°54' E	NT	18	15	0.9739	0.00432	3.22392	2(5)	5	1.0000	0.00763	6.32526
8	Wenzhou	Zhejiang	28°0' N, 120°42' E	WZ	30	13	0.8667	0.01339	9.99900	19(47)	39	0.9898	0.00724	6.08824
9	Xiapu	Fujian	26°53' N, 120°0' E	XP	11	7	0.9091	0.00215	1.60789	8(21)	18	0.9714	0.00722	6.04409
10	Xiamen	Fujian	24°29' N, 118°5' E	XM	19	12	0.9298	0.00551	4.11775	12(45)	42	0.9970	0.00644	5.38438
11	Zhangpu	Fujian	24°7' N, 117°37' E	ZP	12	6	0.7576	0.00171	1.27902	9(23)	22	0.9960	0.00586	4.93081
12	Shantou	Guangdong	23°21' N, 116°41' E	ST	14	8	0.8571	0.00487	3.63484	2(6)	6	1.0000	0.00526	4.37433
13	Yangjiang	Guangdong	21°51' N, 111°59' E	YJ	4	2	0.5000	0.00067	0.50165	3(8)	7	0.9643	0.00422	3.49438
14	Zhanjiang	Guangdong	21°16' N, 110°21' E	ZJ	28	12	0.8519	0.00483	3.61025	8(21)	18	0.9810	0.00509	4.25208
15	Xuwen	Guangdong	20°19' N, 110°10' E	XW	16	11	0.9333	0.00537	4.01209	6(20)	16	0.9737	0.00614	5.12029
16	Qishui	Guangdong	20°46' N, 109°46' E	QS	11	9	0.9636	0.00555	4.14365	2(5)	5	1.0000	0.00393	3.26097
17	Caotan	Guangdong	21°16' N, 109°47' E	CT	8	5	0.7857	0.00473	3.52944	2(8)	8	1.0000	0.00434	3.62715
18	Beihai	Guangxi	21°29' N, 109°7' E	BH	23	14	0.9289	0.00596	4.45226	6(18)	17	0.9935	0.00585	4.88185
19	Qinzhou	Guangxi	21°59' N, 108°39' E	QZ	11	8	0.9273	0.00496	3.70596	3(11)	10	0.9818	0.00566	4.74695
20	Fangchenggang	Guangxi	21°41' N, 108°21' E	FG	10	5	0.7556	0.00423	3.15783	2(7)	7	1.0000	0.00457	3.79816
21	Lingao	Hainan	19°48' N, 109°43' E	LG	12	5	0.7424	0.00430	3.20805	7(22)	19	0.9827	0.00760	6.40287
22	Haiphong	Vietnam	20°56' N, 106°39' E	HP	13	5	0.7949	0.00425	3.17785	9(17)	14	0.9779	0.00598	4.99641
Total					305	97	0.9483	0.03364	25.12570	120 (345)	286	0.9968	0.00734	6.31526

### DNA extraction, PCR amplification, cloning, and sequencing

Genomic DNA was extracted from the ethanol-preserved adductor muscle using an improved phenol-chloroform procedure, which was described by Li et al [48]. A fragment of COI gene was amplified for all samples, while a fragment of ITS gene was only amplified for partial samples to verify the results of COI (Table 1). Polymerase chain reaction (PCR) was carried out in a 50 μL volume containing 2 U Taq DNA polymerase, 100 ng template DNA, 1 μM each primer, 0.2 mM of each dNTP, 1×PCR buffer, 2 mM MgCl_2_, and 4% DMSO. For a fragment of COI gene amplifications, we used the primers: LCO1490 5’-ATTATTCAGAACCAATCATAAAGATATTGG-3’ and HCO2198 5’-TGTAGGAATAGCAATAATAAAAGTTAC-3’ [44]. For a fragment of ITS gene amplifications, we used the primers: PEF-10 5’-TAGAGGAAGGAGAAGTCGTAACAAGG-3’ and 5.8R 5’-CAAKRTGCGTTCRARRTGTCGATGWTCA-3’ [49]. PCR was performed in a GeneAmp^®^ 9700 PCR System (Applied Biosystems, Foster City, CA, USA) under the following conditions: an initial denaturation for 3 min at 94°C, followed by 35 cycles of 40 s at 94°C, 40 s at 48°C in COI or 40°C in ITS, 40s at 72°C, and completed with a final extension for 5 min at 72°C.

For COI, PCR products were directly sequenced using respective primers on an ABI 3730 automated sequencer. For ITS, PCR products were purified by DNA gel extraction kit UNIQ-10 following the recommended protocol, then were cloned into cloning vector pEASY-T1 (TaKaRa). For each individual, we selected two to five clones to sequence by M13 primers.

### Sequence analysis

Sequences of COI and ITS were assembled and checked by SeqMan in DNASTAR software. The consensus sequences were then aligned with BioEdit [50] using ClustalW [51] under default settings. Sequences were cut into the same length using MEGA 6.0 [52]. Software RDP4 [53] was used to detect genetic recombination by the default parameter settings within ITS sequences. Haplotypes of sequences were determined with the software program DnaSP v5 [54], and sites with gaps/missing in the ITS-1 sequences were considered. All sequences of haplotypes were deposited in GenBank database (Accession numbers: KY318078-KY318174, KY318176-KY318461). The program Arlequin 3.5 [55] was used to analyze molecular diversity indices including haplotype diversity (h), nucleotide diversity (Π), and mean number of pairwise differences (k). For the net average genetic distance between major lineages, the Kimura 2-parameter (K2P) model [56], pairwise deletion of gaps data, and “Transitions + Transversions” options were used to calculate in MEGA 6.0 [52].

### Phylogenetic analyses

The neighbor-joining (NJ) tree with bootstrap analysis (1000 pseudoreplicates) was constructed using MEGA 6.0, and parameters setting was same as distance analyses. Bayesian inference (BI) was performed in MrBayes 3.2.0 [57], in which the Markov-chain Monte Carlo (MCMC) search was run with four chains for 50 million generations with a sampling frequency of 1/1000 trees. Substitution models were inferred using jModeltest 0.1.1 [58] using the Akaike information criterion (AIC). *M*. *lamarckii* (KY318175) was used as outgroup.

### Genetic diversity and population structure

Hierarchical analyses of molecular variance (AMOVA) [59] were performed using Arlequin 3.5 on both COI and ITS data set to evaluate population structure. The variance components, sum of squares and Φ statistics were calculated between lineages, among populations within lineages and within populations, respectively. The significance of Φ-statistic analogs was evaluated with 10000 random permutations, and the Tamura-Nei model was selected to correct for multiple substitutions because the HKY+I model is not implemented in Arlequin. Haplotype network showing the genetic relationships was generated with PopART 1.7 [60] at the provincial level based on COI gene.

Pairwise Φ_ST_ values among populations within lineages were evaluated using COI gene by Arlequin 3.5, and the statistical significance was tested by 10000 random permutations. Mantel tests to test the relationship between genetic distances and geographic distances were performed on the Isolation by Distance Web Service (IBDWS at http://ibdws.sdsu.edu/~ibdws/) [61] with 10,000 permutations using COI sequences.

To infer if there had been a past population expansion or selective sweep of *M*. *petechialis*, Fu’s Fs [62] and Tajima’s D [63] statistics along with their statistical significance (1000 permutations) were carried out using DnaSP v5. For the purposes of eliminating the possibility of a selective sweep, the McDonald-Kreitman test [64] was conducted in DnaSP v5 using *M*. *lusoria* as outgroup species. Because ITS-1 was not a protein-coding gene, only COI sequences were analyzed for the McDonald-Kreitman test. In cases where expansion was evident based on neutrality statistics, statistics in mismatch distributions were performed in DnaSPv5 to test whether a sudden population expansion occurred in the history. Sum of squared deviation (SSD) [65] and Harpending’s raggedness index (RI) [66] were calculated in Arlequin 3.5.

### Divergence time estimation

Divergence time between the major distinct lineages was estimated in Beast v1.7.5 software [67] only on the more stable COI marker with a calibrated molecular clock and HKY+I models. We supposed an uncorrelated lognormal distribution for the clock and speciation: Yule Process as the tree prior. Analyses were run for 10 million iterations and sampled every 1000 iterations with a final burn-in of 10%. The lack of a fossil record for *M*. *petechialis* precludes an estimate of a species-specific molecular clock, which will influence any estimates of divergence time. Molecular clocks have been estimated for the COI gene in several bivalves such as *Cyclina sinensis* (0.7–2.4%/Myr) [31], *Ruditapes philippinarum* (0.9–3.35%/Myr) [32], *Tegillarca granosa* (2–2.4%/Myr) [33] and *Atrina pectinata* (2.4%/Myr) [68], but the rates vary widely. In our study, we used molluscan specific mtCOI divergence rates calibrated for bivalve (1%/Myr) [19, 69] and gastropoda (2%/Myr) [70, 71] to estimate the divergence time between two lineages. Although large variance might exist in the divergence rate of 1%/Myr to 2%/Myr for *M*. *petechialis*, the rate was used to estimate a rough time for elucidating phylogeographic hypotheses. The mean substitution rates defined in our analyses were obtained by dividing the mean sequence divergence rates by two [72]. Convergence diagnostics were conducted in Tracer v1.5 [73] and the effective sample size (ESS) for each parameter exceeded 200. TreeAnnotater v1.7.5 was used to generate the maximum credibility tree.

## Results

### Genetic diversity

The aligned COI and ITS-1 gene sequences were obtained with lengths of 747 bp and 860 bp, respectively. The obtained 305 COI gene sequences without indels contained 112 variable sites and 68 parsimony informative sites, yielding 97 haplotypes, of which 72 (74.2%) haplotypes were singletons, being represented by a single sequence in the sample. Wenzhou population showed the highest values of nucleotide diversity (0.01339 ± 0.00701) and mean number of pairwise differences (9.99900 ± 4.70302) because lineage overlap was observed in this population. Overall values of COI gene for haplotype and nucleotide diversities were 0.9483 ± 0.0054 and 0.03364 ± 0.01638, respectively. The obtained 345 ITS-1 gene sequences contained 194 variable sites and 65 parsimony informative sites yielding 286 haplotypes, in which 260 (90.9%) haplotypes were singletons. Overall values of ITS-1 gene for haplotype and nucleotide diversities were 0.9968 ± 0.0009 and 0.00734 ± 0.00386. No genetic recombination was detected within ITS-1 sequences. Overall, values of haplotype diversity, nucleotide diversity and mean number of pairwise differences for ITS-1 gene were higher than those of COI gene (Table 1).

### Phylogenetic analyses

**Fig 2:**
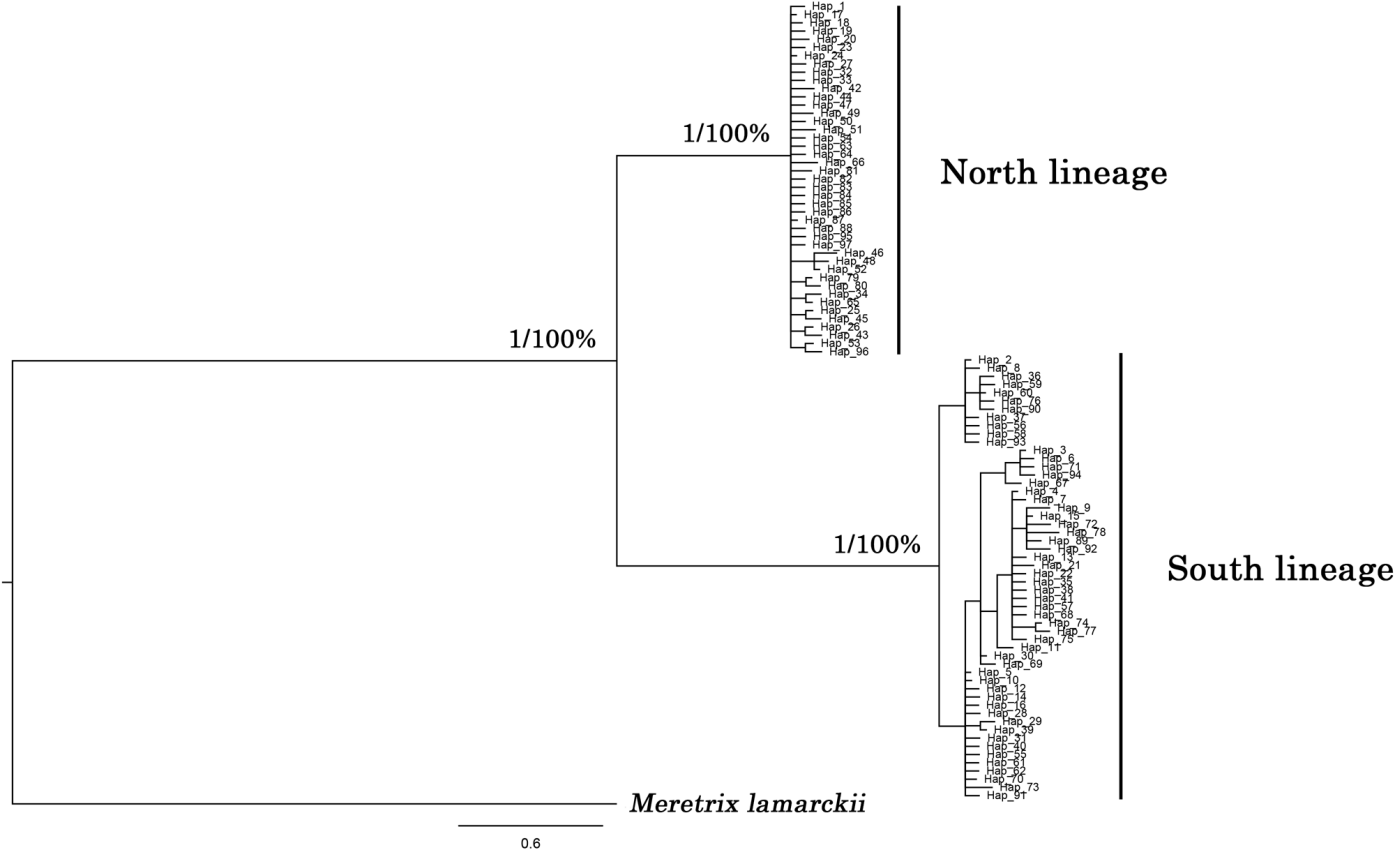
Phylogenetic hypothesis based on COI sequences represented by Bayesian inference method. Bootstrap values were represented near the branches (BI and NJ bootstrap values, respectively).

Phylogenetic reconstruction with the COI gene based on NJ and BI methods separated *M*. *petechialis* into two lineages with high nodal support (Fig 2), which were identified as North and South lineages, each corresponding to one of the biogeographic areas. North lineage included samples from Bohai Sea, Yellow Sea and East China Sea, while samples of South lineage were from East China Sea and South China Sea. The overlap zone was observed in Zhejiang and Fujian provinces. But no obvious difference was found between two lineages in phylogenetic reconstruction with the ITS-1 gene. The net average genetic distance (± SD) between lineages was 5.85 ± 0.95% for COI and 0.08 ± 0.03% for ITS-1.

### Genetic diversity and population structure

When populations were grouped by sea basins, for COI gene, hierarchical analyses of AMOVA (Table 2) indicated that the most of the total genetic variation (93.22%) was from differences between two lineages (*P* < 0.01). A smaller (5.98%, *P*<0.01) amount of genetic diversity occurred within populations, and the smallest (0.80%), yet significant (*P* < 0.01), of the total variation was present among populations within lineages. For ITS-1 gene, the result showed that the most of the total genetic variation (84.20%, *P*<0.01) was apportioned within populations, followed by a smaller (9.00%, *P*<0.01) amount occurred between groups, and only a small part (6.81%, *P*<0.01) among populations within groups.

**Table 2:** Results from analysis of molecular variance (AMOVA) of population structure. Significant *P* values are bolded.

Marker/Grouping	Source of variation	d.f.	Sum of squares	Φ-Statistics	% of variation	*P* value
COI	Between lineages	1	3402.349	Φ_CT_ = 0.93216	93.22	**< 0.0001**
	Among populations within lineages	27	92.298	Φ_SC_ = 0.11862	0.80	**< 0.0001**
	Within populations	282	399.838	Φ_ST_ = 0.94020	5.98	**< 0.0001**
ITS-1	Between lineages	1	57.965	Φ_CT_ = 0.08997	9.00	**< 0.0001**
	Among populations within lineages	25	139.133	Φ_SC_ = 0.07481	6.81	**< 0.0001**
	Within populations	318	889.127	Φ_ST_ = 0.15805	84.20	**< 0.0001**

**Fig 3:**
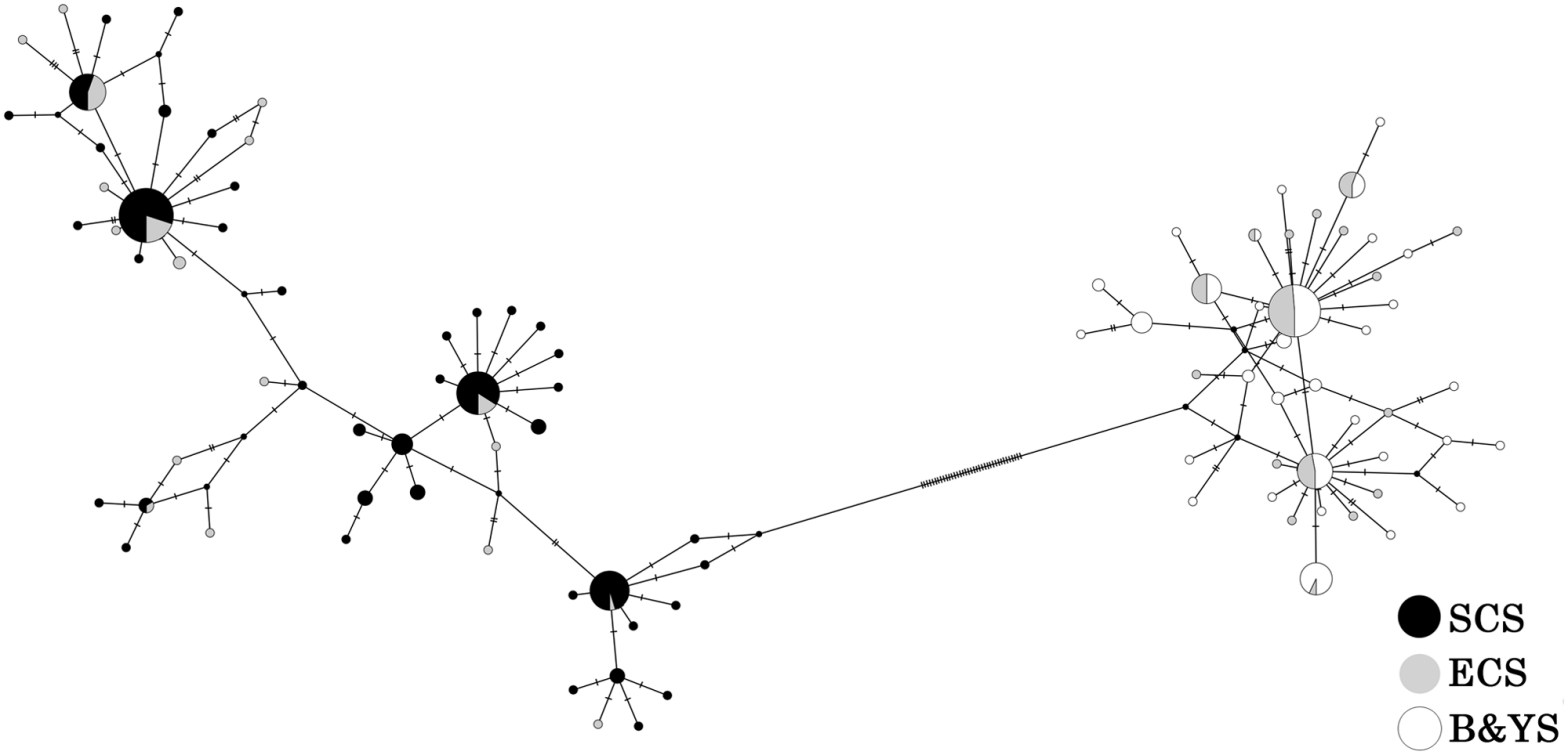
Network of *M*. *petechialis* using COI data. The pie charts refer to the haplotypes. B&YS: Bohai and Yellow Sea; ECS: East China Sea; SCS: South China Sea.

Based on the COI, two haplogroups were formed in the network (Fig 3), which was consistent with the BI and NJ trees. The topology of haplotype network was characterized by multiple star-like type. Significant overlaps between two lineages were observed in 23° to 29° N, where is located at the adjacent area of the ECS and SCS.

**Fig 4:**
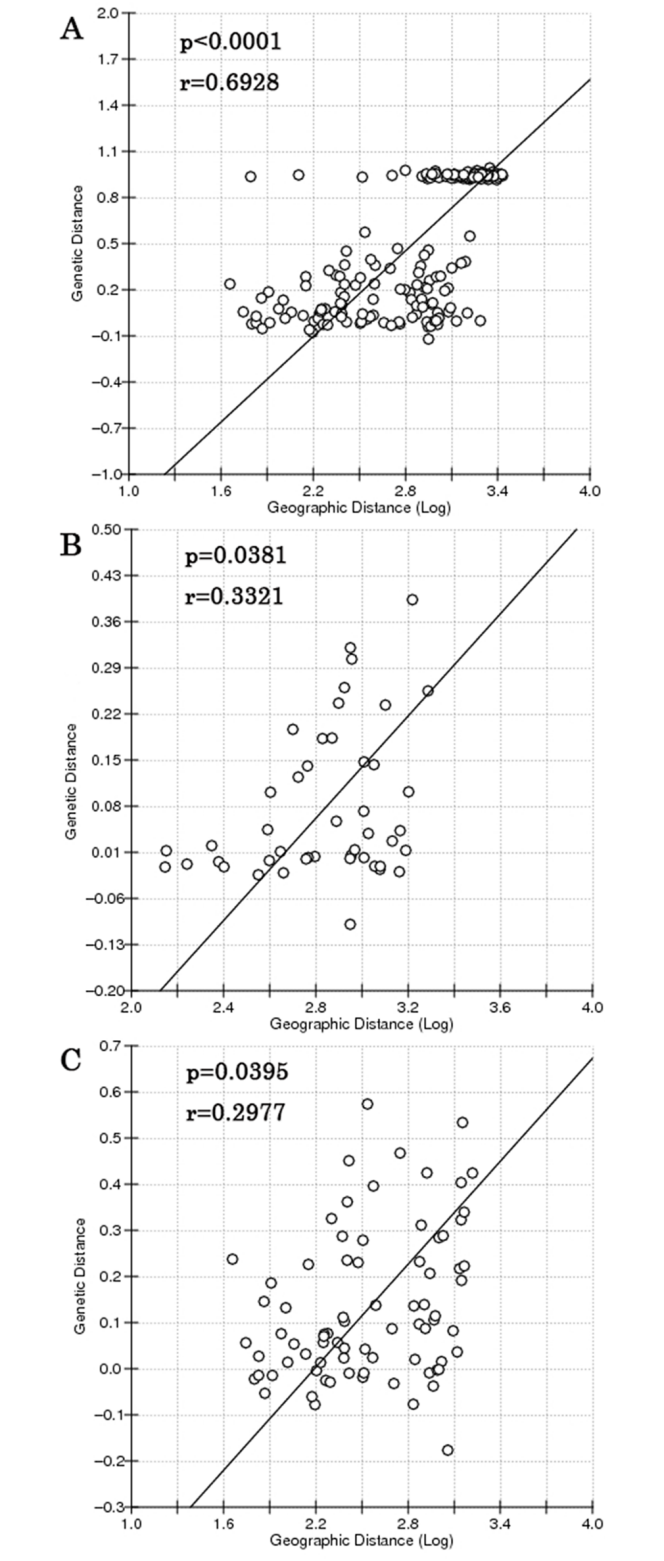
Relationship between genetic vs. geographical distance (log-transformed) in *M*. *petechialis*. A, all China populations except Wenzhou; B, the North populations; C, the South populations.

Pairwise Φ_ST_ values based on COI sequences among populations within lineages were shown in Tables 3 and 4. For North lineages, Changyi and Weihai populations were found significantly different from others except for Busan. For South lineages, Xuwen, Qishui, Qinzhou and Haiphong populations located in Beibu Gulf, were remarkably different from Wenzhou, Xiamen, Shantou and Zhanjiang populations. The IBD analysis displayed a significant relationship between genetic distances (F_ST_) and geographic distances (Fig 4. All populations: r = 0.6928, p<0.0001; North populations: r = 0.3321, p = 0.0381; South populations: r = 0.2977, p = 0.0395).

**Table 3:** Pairwise Φ_ST_ values based on COI (below diagonal) and associated P values (below diagonal) after Bonferroni correction between populations within North lineage. Significant P values (P < 0.05) after 10,000 permutation are bolded.

	BS	PJ	WH	CY	QD	GY	NT	WZ	XP	ZP
BS		**0.0046**	0.2141	0.7948	**0.0200**	0.0904	**0.0354**	**0.0028**	0.0571	**0.0108**
PJ	0.4573		**0.0001**	**0.0000**	0.7327	0.0953	0.9356	0.1211	0.0935	0.4571
WH	0.1812	0.3589		**0.0253**	**0.0006**	**0.0020**	**0.0016**	**0.0000**	**0.0103**	**0.0002**
CY	-0.1210	0.3390	0.1821		**0.0002**	**0.0099**	**0.0003**	**0.0000**	**0.0078**	**0.0003**
QD	0.3538	-0.0224	0.2943	0.2850		0.0796	0.6825	0.0578	0.0649	0.5826
GY	0.2602	0.0398	0.2386	0.1551	0.0464		0.2788	0.0995	0.8959	0.0652
NT	0.2141	-0.0260	0.2008	0.2034	-0.0142	0.0082		0.2433	0.5244	0.6698
WZ	-0.0478	0.0246	0.3641	0.3311	0.0387	0.0272	0.0091		0.5224	0.6345
XP	0.3415	0.0484	0.2105	0.1934	0.0505	-0.0420	-0.0072	-0.0093		0.1432
ZP	0.5484	-0.0018	0.3831	0.3727	-0.0038	0.0558	-0.0133	-0.0143	0.0342	

**Table 4:** Pairwise Φ_ST_ values based on COI (below diagonal) and associated *P* values (below diagonal) after Bonferroni correction between populations within South lineage. Significant *P* values (*P* < 0.05) after 10,000 permutation are bolded.

	WZ	XM	ST	YJ	ZJ	XW	QS	CT	BH	QZ	FG	LG	HP
WZ		0.1781	0.6513	0.7153	0.2701	**0.0351**	**0.0076**	0.0333	0.0681	**0.0032**	**0.0394**	0.0592	**0.0045**
XM	0.0867		**0.0392**	0.0878	0.5316	**0.0186**	**0.0201**	0.7252	0.4277	**0.0004**	0.2608	0.3923	0.0550
ST	-0.0770	0.0764		0.5758	0.1839	**0.0032**	**0.0004**	0.0811	**0.0072**	**0.0001**	**0.0073**	0.0577	**0.0012**
YJ	-0.1766	0.1361	-0.0321		0.1418	**0.0109**	**0.0017**	0.0733	**0.0166**	**0.0007**	**0.0186**	**0.0325**	**0.0017**
ZJ	0.0363	-0.0091	0.0201	0.0748		**0.0090**	**0.0037**	0.5197	0.1067	**0.0000**	0.1107	0.4055	**0.0129**
XW	0.3226	0.1058	0.2322	0.3617	0.1321		0.4904	0.1548	0.2536	0.1611	0.0600	0.2109	0.2458
QS	0.4036	0.1146	0.3112	0.4508	0.1857	-0.0144		0.1634	0.2694	0.1512	0.1143	0.0925	0.5259
CT	0.2169	-0.0376	0.0967	0.2871	-0.0223	0.0535	0.0561		0.8372	**0.0248**	0.7372	0.7859	0.4511
BH	0.1913	-0.003	0.1390	0.2302	0.0318	0.0131	0.0139	-0.0533		**0.0127**	0.5056	0.6566	0.4758
QZ	0.5337	0.2840	0.4245	0.5737	0.3254	0.0445	0.0568	0.2261	0.1457		**0.0138**	**0.0110**	0.0758
FG	0.3394	0.0154	0.2066	0.3960	0.0568	0.1027	0.0702	-0.0779	-0.0146	0.2375		0.2507	0.4875
LG	0.2227	-0.0016	0.0872	0.2784	-0.0041	0.0270	0.0758	-0.0606	-0.0252	0.2351	0.0231		0.1895
HP	0.4241	0.0822	0.2883	0.4674	0.1376	0.0239	-0.0185	-0.0093	-0.0098	0.1113	-0.0291	0.0423	

**Fig 5:**
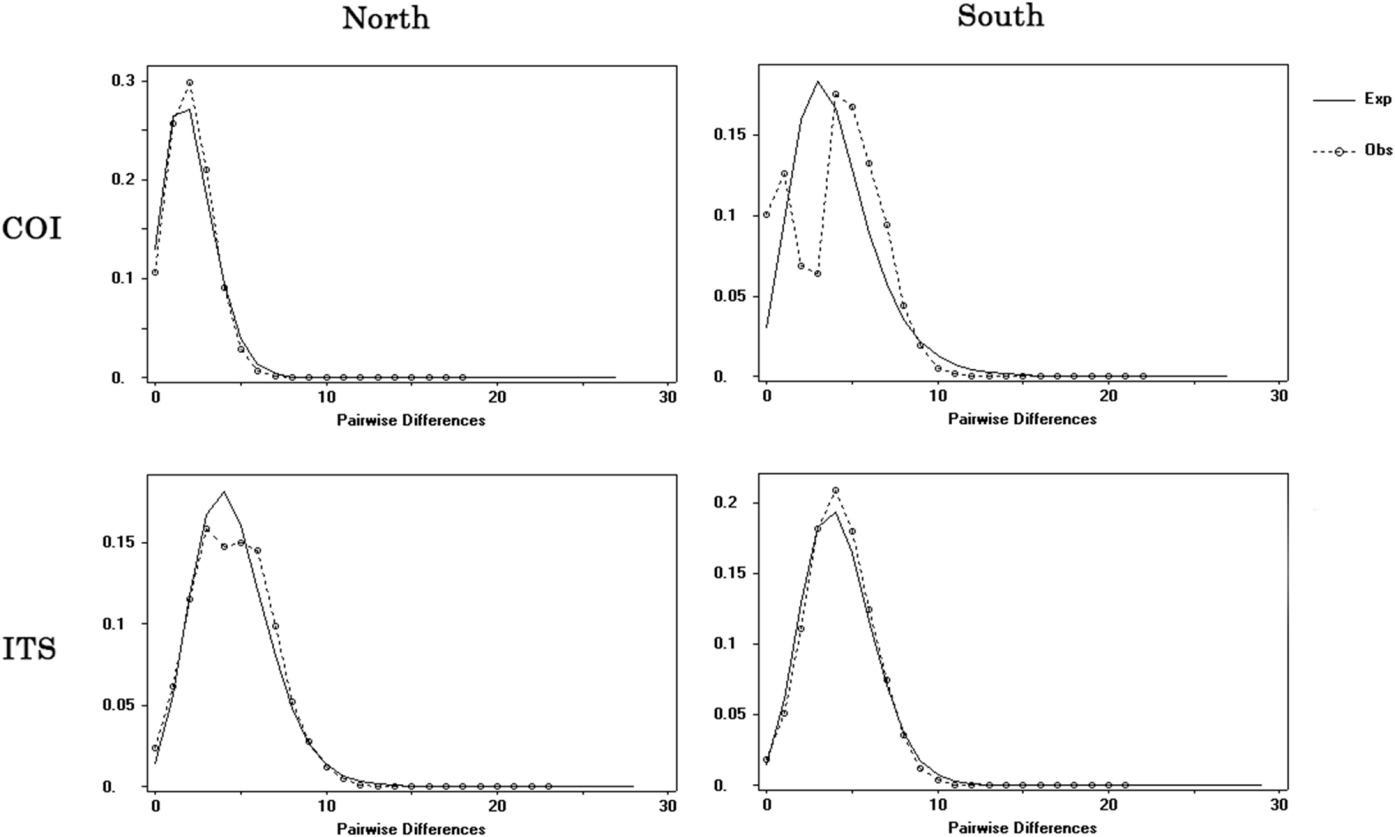
Mismatch distributions for two lineages. Dotted lines with circles represent the observed frequency of pairwise differences, whereas the solid lines show the expected values under the sudden population expansion model.

The McDonald and Kreitman test demonstrated no significant difference (NI = 1.329, Fisher’ s exact test P-value = 0.764), ruling out the possible of the selective sweep. Estimates of neutral tests for two lineages conducted on both COI and ITS data indicated population expansion by significant negative values of Fu’ Fs and Tajima's D statistics (Table 5). The observed mismatch distributions of two lineages based on COI and ITS data (except for Southern lineage on COI) uniformly displayed unimodal distributions, which fit well with the model of sudden expansion (Fig 5). The RI and SSD for two lineages all matched the null hypothesis of sudden expansion model with nonsignificant values (Table 5). Via an inspection of the network of lineage south (Fig 3), two haplogroups could lead to the bimodal distribution.

**Table 5:** Estimates of neutral tests for population expansion and the mismatch distribution parameters RI and SSD of each lineage. Significant *P* values for Fu’ Fs and Tajima's D (*P* <0.05) and nonsignificant values for RI and SSD (*P* >0.05) are bolded.

		Fu' F_S_	*P*	Tajima	*P*	SSD	*P*	RI	*P*
Lineage North	COI	-27.27941	**0.00000**	-2.28510	**0.00010**	0.00172	**0.20000**	0.05066	**0.15000**
	ITS-1	-24.02432	**0.00080**	-1.95655	**0.00380**	0.00058	**0.87000**	0.00118	**1.00000**
Lineage South	COI	-25.65390	**0.00000**	-1.80375	**0.00860**	0.00997	**0.24000**	0.02242	**0.35000**
	ITS-1	-23.90534	**0.00160**	-2.37893	**0.00010**	0.00035	**0.97000**	0.00155	**0.99000**

### Divergence time estimation

**Fig 6:**
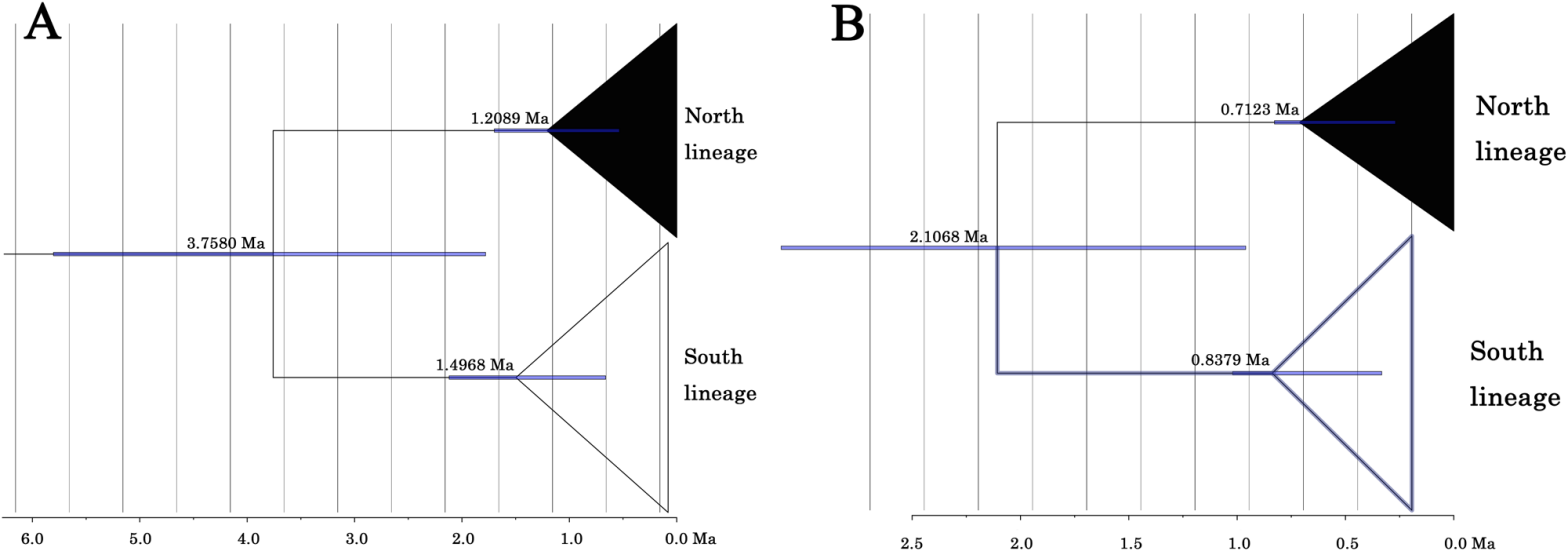
BEAST divergence time estimation of *M*. *petechialis* using calibrated molecular clocks method. Divergence rates: A, 1%/Myr; B, 2%/Myr. Node ages were represented near the branches. Blue bars on branch nodes indicate 95% Highest Posterior Density age intervals. Timescales in million years before present (Ma).

Results of the COI divergence time estimations for *M*. *petechialis* are shown in Fig 6. The divergence age estimate between North and South lineages was approximately 2.1–3.8 Ma, corresponding to the early Pleistocene to late Pliocene. The time of origin of North and South lineages were about 0.7–1.2 Ma and 0.8–1.5 Ma respectively, dating back to Pleistocene.

## Discussion

### Phylogeographical patterns

Based on the network relationships and phylogenetic reconstruction for COI markers, significant population differentiation were observed between northern (ECS) and southern groups (SCS), and overlapped at the adjacent area of the ECS and SCS (Fig 1). In addition, genetic differentiation between two lineages accounted for a large proportion according to the AMOVA analysis. Therefore, we concluded that phylogeographical pattern of *M*. *petechialis* was connected to the historical isolation of marginal seas, which is consistent with the observations in mitten crab *Eriocheir sensu* stricto [74], marine fish (*Chelon haematocheilus* [75] and *Bostrychus sinensis* [76]) and bivalves (*Cyclina sinensis* [31] and *Tegillarca granosa* [33]). The divergence time between two lineages dated back to 2.1–3.8 Ma, generally near to early glaciations intensified time and the start time that sea level major drops 60 to 120 m (2.5 Ma) [8,77]. Because of the sea level falling, ECS and SCS were isolated, so a physical barrier was created for *M*. *petechialis*, giving rise to divergent lineages. The divergence time in our study is similar with two subspecies of oyster (2.0–3.6 Ma) [43] and two species in the mitten crab (2.24 Ma) [78]. Above all, in the northwestern Pacific region, historical glaciation during Pleistocene played an effective role in generating intraspecific genetic divergences [25]. In postglacial period, sea level rising and land bridge disappearing contributed to rapid population expansion of intertidal species and secondary contact at the adjacent areas [16,79,80]. The signal of the demographic expansion of *M*. *petechialis* was also detected through star-like type of network topology, mismatch distribution analysis and neutrality tests.

One major cooling period was occurred in the 2.2–1.0 Ma, which are reflected in the increasing SST gradient in the subtropical area, where SST cooled significantly (decreased by 3.3–5.4°C and 1.0–2.1°C in winter and summer respectively), while SST showed little or no cooling (decreased by 0.9°C and 0.6°C in winter and summer respectively) in the tropical western Pacific [81]. SST gradient between the ECS located in the subtropical area and the SCS located in the tropical area might lead to genetic divergence combined with glacial isolation. Furthermore, SST possibly plays an important role in maintaining phylogeographical pattern of *M*. *petechialis*. Firstly, the boundary of two *M*. *petechialis* groups is approximately near to Taiwan Strait. In the northwestern Pacific, the Tropic of Cancer is the boundary between the warm-temperate and tropical regions, and 20°C isotherm of annual mean temperature running through the Taiwan Strait, which is a major biogeographic boundary for many species [82]. Secondly, analogous to two lineages of *Mugil cephalus* [17], the distribution range of northern group is consistent with gradients of the cold waters from the Subei Coastal Current (SCC) flowing southward along the ECS coast, while the southern group appeared be restricted to the warm water of the China Coastal Current (CCC) flowing from the SCS into the ECS. Thirdly, because of the temperature difference from the north to south, northern populations have a later reproduction period. These phenomena indicated that SST might limit the dispersal of *M*. *petechialis*. Moreover, from 1982, SST have continued to rise [83,84], which will potentially promote species to migrate northward along Chinese coastline [29].

Similarly, *Tegillarca granosa* and *Cyclina sinensis*, plankton larval duration of *M*. *petechialis* is about 5–8 days, however, *M*. *petechialis* migrates from mid-tidal region to low-tidal region along with tidal currents extending from late May to late June and mid-September to late-September. This specific feature may increase opportunity for gene flow among different populations. During the breeding season of *M*. *petechialis* (spring and summer), the China Coast Current with a common velocity of 20 cm/s [34] flowing northward, transported pelagic larvae from SCS to ECS. Overlapping phenomenon was found at the adjacent area of the ECS and SCS (23° to 29° N) because of secondary contact, which might be promoted by the connectivity of China Coast Current [25]. Significant relationship was found between genetic distances (**Φ**_ST_) and geographic distances when the North and South populations were analyzed respectively, after fitting IBD model. In addition, some pairwise Φ_ST_ values were significant between populations within each lineage. For North lineage, the genetic difference between some Bohai Sea populations (Changyi and Weihai) and East Sea populations were statistically significant. For South lineage, some populations located in Beibu Gulf (Xuwen, Qishui, Qinzhou and Haiphong) were significantly divergent from Wenzhou, Xiamen, Shantou and Zhanjiang populations. This is most likely attributed to the low-dispersal ability of the clam and the existence of small but persistent reciprocal flow and rotating flow along coastal areas, which impede the exposure of larvae to currents and their ability to be transported effectively [31].

He et al.’s [85]study indicates that the South China Sea should be considered to be a colonization origin of *Periophthalmus modestus* because of an older coalescence time, a putative ancestral haplotype, higher genetic diversity, more proportion of private haplotypes and higher species diversity of the genus *Periophthalmus*. Similarly, we proposed that South China Sea is the earlier refuge for *M*. *petechialis*. First of all, north lineage originated from refugium in the Okinawa Trough about 0.7 Ma, whereas South lineage derived from refugium in South China Sea about 0.8 Ma. Although they are approximate times, the relative coalescence times of two populations are reasonable. Moreover, the portion of private haplotypes from South lineage (75.9%) was higher than that from North lineage (72.1%), which supported the South China Sea was the colonization origin [86]. In addition, haplotype and nucleotide diversity (h: 0.8996, Π: 0.00543) of South lineage had higher values compared with Northern lineage (h: 0.8939, Π: 0.00274). Generally speaking, older colonized regions are expected to exhibit higher genetic diversity due to the longer evolutionary history [87]. Finally, although the bivalve genus *Meretrix* Lamarck, 1799 are broadly distributed in the West Pacific and the Indian Ocean, *M*. *petechialis* is the only *Meretrix* species in the ECS regions.

### The discrepancy between mitochondrial and nuclear topologies

MtCOI gene analysis separated *M*. *petechialis* into two lineages, while nrITS data showed a slightly different topology. The net average genetic distance (± SD) between lineages was 5.85 ± 0.95% for COI, but only 0.08 ± 0.03% for ITS. Result of AMOVA also displayed that nrITS were not significantly differentiated between groups. This inconsistent pattern has also been found in black-throated tits *Aegithalos concinnus* [88] and the pen shell *Atrina pectinata* [89]. A large proportion of cases that discordance between mitochondrial and nuclear data likely appeared following geographic isolation and secondary contact, which attributed to incomplete lineage sorting and post-glacial introgression, respectively [47]. However, they are difficult to be distinguished [90]. COI gene will have a four times faster coalescence time than that of ITS gene [1], because this rate is inversely proportional to the effective population size [91] (a fourfold smaller effective population size [92]). In our study, the divergence time between two lineages dates back to a relatively young divergence (Pleistocene). It is reasonable that ITS gene keep homogeneous and will have longer time to spit, while COI gene have achieved coalescent. Furthermore, under incomplete lineage sorting, no discernible biogeographic pattern among populations is expected to be found [91]. In our study, phylogenetic reconstruction with the COI gene separated *M*. *petechialis* into two lineages, but no obvious difference and discernible geographic pattern was found between the two lineages using the ITS gene. We also found samples from different mtDNA lineages shared the same haplotype for ITS gene, for example, samples from Busan, Beihai and Caotan shared the same haplotype. Therefore, incomplete lineage sorting is more likely to be the reason of the discepancy between mitochondrial and nuclear topologies.

## Conclusion

To better elucidate phylogeographic pattern of *M*. *petechialis*, we sequenced the COI and ITS1 genes from 311 individuals sampled from 22 locations along the coastal areas of China, Korea and Vietnam. The results provided evidence for strong genetic divergence between two lineages, which might be attributed to variance of sea level changes and SST gradients. Here, the combinatory use of nuclear ITS gene and the mitochondrial COI gene helped elucidate the phylogeographical pattern of *M*. *petechialis*, such as population structure and population expansion analyses. With the objectives of getting more detailed information about phylogeography of *M*. *petechialis* with higher genetic resolution, analyses with multiple nuclear genes would be conducted.
